# Inhibition of N^ε^‐(carboxyethyl)lysine and N^ε^‐(carboxymethyl)lysine formation in beef, chicken, and fish meat: A comparative study of oven frying and air frying with a marinade‐containing *Micromeria fruticosa*

**DOI:** 10.1002/fsn3.4276

**Published:** 2024-06-14

**Authors:** Serap Kılıç Altun, Mehmet Emin Aydemir, Kasım Takım, Mustafa Abdullah Yilmaz

**Affiliations:** ^1^ Department of Food Hygiene and Technology, Faculty of Veterinary Medicine Harran University Şanlıurfa Turkey; ^2^ Department of Basic Sciences, Faculty of Veterinary Harran University Şanlıurfa Turkey; ^3^ Department of Analytical Chemistry, Faculty of Pharmacy Dicle University Diyarbakır Turkey

**Keywords:** AGEs, CEL, CML, meat processing, *Micromeria fruticosa*, TBARS

## Abstract

The objective of this study was to assess the impact of marinating beef, chicken, and fish with *Micromeria fruticosa* (*M. fruticosa*) on the inhibition of N^ε^‐(carboxyethyl)lysine (CEL) and N^ε^‐(carboxymethyl)lysine (CML). Furthermore, our objective was to examine how different cooking techniques, temperatures, and durations affect the creation of CEL and CML in these meat products. The study began with the characterization of *M. fruticosa*. Subsequently, meat samples were marinated using an *M. fruticosa*‐containing marinade and stored at 4 ± 1°C for 24 h. Following storage, the meats underwent cooking in an oven at 200°C for 12 min and in an air fryer at 250°C for 8 min. Subsequently, pH, color, thiobarbituric acid reactive substances (TBARS), as well as CEL and CML analyses were conducted. *M. fruticosa* had high levels of biological activity and bioactive content. Moreover, increasing the *M. fruticosa* ratio in the marinade demonstrated a reduction in TBARS, CML, and CEL formation. This study concludes that *M. fruticosa* can be effectively used as a marinade component for meat, inhibiting the formation of CEL and CML. In conclusion, this research underscores the significant potential of *M. fruticosa* in reducing the synthesis of advanced glycation end products (AGEs) during meat processing. These results not only enhance our comprehension of the complex relationship between plant extracts and meat quality but also present encouraging prospects for fostering healthier and safer cooking methods.

## Graphical abstract

This article offers a natural solution to inhibit advanced glycation end products (AGEs) formed during cooking of meats. It also discusses the health effects of cooking meat with air fryer, a new cooking technique. The aim of this study is to determine the effect of different cooking methods, cooking temperatures, and durations on the inhibition of N^ε^‐(carboxymethyl)lysine (CML) and N^ε^‐(carboxyethyl)lysine (CEL) in red meat, fish meat, and poultry meat marinated with varying concentrations of *Micromeria fruticosa*.

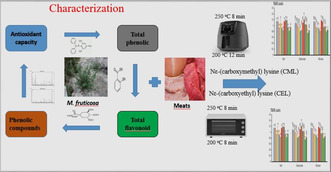

## 1 INTRODUCTION

The Maillard reaction, a complex sequence of chemical reactions involving amino acid residues and reducing sugars, is pivotal in generating advanced glycation end products (AGEs) during food preparation (Hellwig et al., 2022). AGEs constitute a class of potentially detrimental compounds associated with the development of various disorders. Dietary intake of AGEs has been identified as a significant contributor to the body's pool of AGEs, correlating with heightened health risks (Dong et al., 2023; Uribarri et al., 2010). Because of their high quantities of total protein and fat, meat products have been identified as a substantial dietary source of AGEs. Two well‐characterized AGEs, N^ε^‐carboxyethyl‐Lysine (CEL) and N^ε^‐carboxymethyl‐Lysine (CML), have been employed as AGE indicators in food (Chen et al., 2022). Understanding the mechanisms that influence CEL and CML development in meat products is critical for identifying potential health hazards and developing appropriate mitigation methods. Recent studies have looked into numerous aspects of AGE development in meat products (Liu et al., 2022; Niu et al., 2018; Tavares et al., 2018). Recent studies have investigated the development of AGEs in meat products from many aspects and have emphasized that many factors influence CEL and CML levels. Given the effects of AGEs on health, it is important to develop strategies to reduce dietary AGEs to the lowest possible levels and to determine AGE levels in foods.

The production and amount of AGEs in food are impacted by several factors such as the food's ingredients (protein, sugar, fat, carbohydrate, water, and minerals), methods of processing, acidity level, cooking methods, intensity and length of cooking, moisture content, water activity (aw), range and rate of the Maillard reaction (MR), and level of oxidation (Aydemir et al., 2023). It is crucial to emphasize that a number of factors, including storage conditions like pH, temperature, humidity, time, type of packaging, protein content, oil content, and oxidation, clearly affect the amount of AGEs found in food (Yu et al., 2016; Zhu et al., 2020). However, the cooking technique used is the most influential factor affecting AGE formation. Generally, as cooking temperatures increase, the concentration of AGEs in foods also tends to increase.

Foods of animal origin, which are rich in fat and protein, are more prone to AGE formation during storage and heat treatment. CML can form in meat and meat products during storage, and the amount of CML increases during storage (Yu et al., 2016). During the preservation of meat, many di‐carbonyl compounds such as free amino acids as a result of oxidation and hydrolysis of myosin and glyoxal (GO) as a result of lipid oxidation are formed. In addition, carbonyl groups may be formed as a result of protein oxidation depending on the processes applied to the meat in this process (Xu, Ni, et al., 2024). It is emphasized that all of these reactions are effective in the formation of free/bound AGEs during preservation, albeit at low levels. It is also stated that the rate of AGE formation increases in parallel with the cooking method of the meat and the degree and duration of heat applied (Aydemir, Arslan, et al., 2024).

Air frying and oven cooking are popular methods for preparing meat dishes. Especially since the air fryer cooking method is fast, practical, and looks healthy because it uses less oil, it has recently found widespread use (Cano et al., 2022). However, it is crucial to consider the formation of AGEs during these cooking processes. AGEs are harmful compounds produced when proteins or fats react with sugars at elevated temperatures. Research indicates that cooking methods involving dry heat, such as air frying and oven frying, can significantly contribute to the formation of AGEs in meat products (Uribarri et al., 2010).

Another commonly used and effective option, in addition to enhancing cooking techniques, is the incorporation of herbal extracts abundant in polyphenols to reduce the concentration of AGEs. It has been reported by some researchers that marinating meat samples with some antioxidant plants or their extracts before cooking prevents the formation of AGEs (Aydemir, Arslan, et al., 2024; Zhu et al., 2021). *Micromeria fruticosa* (*M. fruticosa*), also known as Lebanese oregano or Turkish stone mint, is an aromatic herb popular in Mediterranean cooking for its distinct flavor and potential health advantages. This herb is native to the Middle East and Mediterranean regions and belongs to the *Lamiaceae* family (Salameh et al., 2020). *M. fruticosa* has attracted attention for its distinctive sensory qualities and potential health‐promoting effects in foods (Sadeq et al., 2021). It is frequently utilized as a seasoning and spice in numerous traditional recipes and culinary dishes, adding a unique and recognizable flavor (Abu‐Reidah et al., 2019). *M. fruticosa* has been recognized for its possible health advantages in addition to its flavor‐enhancing characteristics. The herb contains a high concentration of bioactive components, including phenolic compounds, flavonoids, and essential oils, which have antioxidant, antibacterial, and anti‐inflammatory activities (Abu‐Reidah et al., 2019; Salameh et al., 2020). These bioactive compounds have been associated with a range of health advantages, such as the capacity to combat microbial infections and regulate inflammatory reactions (El Hamaoui et al., 2015).

The aim of this study was to determine the effect of marinating beef, chicken, and fish meat with *M. fruticosa* on the inhibition of CEL and CML and to determine the effect of cooking these meats at different methods (oven and air fryer), temperatures, and times (12 min at 200°C and 8 min at 250°C) on the amount of CEL and CML formation.

## 2 MATERIALS AND METHODS

### 2.1 Material, extraction methods, and characterization techniques

#### 2.1.1 Collection and preparation of *M. fruticosa*

Regional samples of 22 different plants of *M. fruticosa* were collected from wildlife habitats in the mountains of the Uzundere District (40°31′55.85″ N, 41°32′17.95″ E), situated at an altitude of 1027–1050 m above sea level, during the summer of 2022. The leaves were detached from the plant in the field and then air‐dried at room temperature. Following this, it was ground to flour in a spice grinder (GG‐30B‐C‐GELGOOG, China) for about 10 min. It was stored at room temperature until needed for extraction and marination. Water and methanol extracts were prepared for the characterization of floured *M. fruticosa*. For the marinade component, determined concentrations were added directly to the marinade.

#### 2.1.2 Sample extraction

Two separate extraction methods, with methanol and water, were employed to extract *M. fruticosa* leaves, each conducted in three independent replicates. The extraction process involved taking 2 g of *M. fruticosa* into a falcon tube and adding 20 mL of solvent (at a ratio of 1:10), followed by homogenization. The sample was then blended in a double boiler at 300 rpm and maintained at 25°C for 24 h with the container sealed, resulting in green‐hued extracts. The extracts were then filtered through Whatman No. 1 filter paper, and a Heidolph‐Rotary TLR 1000 rotary evaporator was used to evaporate the solvents. Drying was then carried out in an incubator (Nüve EN 400, Turkey). The extracts were kept at 60°C for 24 h and further processing was continued on completely dried extracts. For nondried products (water extracts), this period was extended to 72 h. The amounts of dry extract obtained were then determined (Aydemir, Arslan, et al., 2024).

#### 2.1.3 Total phenolic compounds (TPC) determination

The concentration of TPC in the samples was assessed following the method outlined by Pavun et al. (2018) with slight adjustments. Specifically, 40 μL of the *M. fruticosa* extract was blended with 200 μL of Folin–Ciocalteu reagent (Merck, Germany). Subsequently, 1160 μL of distilled water was added, and the mixture was shaken for 5 min. Following that, 600 μL of a 20% Na2CO3 solution was poured, vortexed for 10 s, and left to shake at 25°C for 2 h. Following the incubation period, the diluted sample was replaced with a blank sample of 10 mL deionized water, and the TPC was measured at 765 nm using a UV spectrometer (CamSpec M550 SBF UV–VIS). The results were represented in milligrams of gallic acid equivalents (GAE) per liter and compared to a standard curve of gallic acid solutions (ranging from 25 to 500 mg/L).

#### 2.1.4 Total flavonoid compounds (TFC) determination

Takım and Işık's (2020) and Aydemir, Arslan, et al.'s (2024) methods were modified to determine the amount of TFC levels in this study. One milliliter of *M. fruticosa* extract, 4 mL of distilled water, and 3 mL of NaNO_2_ were combined in a tube along with different concentrations of a standard quercetin solution (50, 100, 200, 400, and 800 mg/mL). 0.3 mL of AlCl_3_ was added after 5 min. 2 mL of NaOH was added at the 6‐min mark, and distilled water was used to get the total amount down to 10 mL. After the solution was well combined, it was kept for 60 min at 25°C. It was then measured at an absorbance of 510 nm in a UV spectrometer (CamSpec M550 SBF UV–VIS) against a reagent blank. Next, the extract's total flavonoid content was measured in milligrams of quercetin acid equivalent (KE) per gram.

#### 2.1.5 Antioxidant activity determination

Using the methodology suggested by Aydemir, Arslan, et al. (2024), the radical scavenging activity of 1,1‐diphenyl‐2‐picrylhydrazil (DPPH), as an indicator of a plant's capability for antioxidants, was assessed. Fifty microliter of the sample was extracted for each concentration, according to the appropriate limits. After adding 0.8 mL of ethanol, the tubes were vortexed and allowed to sit in the dark at 25°C for half an hour. Then, 0.2 mL of a 1 mM DPPH radical that had been formed in ethanol was added. Following the incubation period, a UV spectrometer (CamSpec M550 SBF UV–VIS) was used to measure the absorbance value at 515 nm in comparison to a control solution. The Trolox equivalent (TE) in grams per gram of dry weight was used to express the antioxidant capability.

The 2,2′‐azinobis (3‐ethylbenzothiazoline‐6‐sulfonic acid) (ABTS) radical scavenging activity, another indicator of plant antioxidant capacity, was determined by modifying the method outlined by Aydemir, Arslan, et al. (2024). Before preparing the ABTS solution, a mixture of the ABTS radical, potassium peroxodisulphate, and ABTS was stored at room temperature for 12–16 h. The resulting solution was diluted with distilled water until the absorbance reached 0.7000 ± 0.020 at 734 nm. For the control, the ABTS solution was added to distilled water in 3 mL cuvettes and incubated at 25°C for 30 min. Results were quantified as micrograms of Trolox equivalent (TE) per gram using a UV spectrometer (CamSpec M550 SBF UV–VIS).

#### 2.1.6 Analysis of the phenolic compound of *M. fruticosa*

An investigation of phenolic compounds using LC–MS/MS was carried out using a Shimadzu HPLC Nexera model and dual MS equipment. The liquid chromatography system was equipped with DGU‐20A3R model degasser, LC‐30 AD model binary pump, CTO‐10ASvp model column oven, and SIL‐30 AC model autosampler. Chromatographic separation was achieved using an Agilent Poroshell 120 EC‐C18 model (150 mm × 2.1 mm, 2.7 μm). The column temperature was maintained at a constant 40°C, and the elution gradient was prepared using mobile phase A (5 mM ammonium formate and 0.1% formic acid in water) and mobile phase B (5 mM ammonium formate and 0.1% formic acid in methanol). The following gradient elution profile was used: 20%–100% B (0–25 min), 100% B (25–35 min), and 20% B (35–45 min). The sample injection volume was set at 4 μL, and the solvent was supplied at a rate of 0.5 mL/min.

A Shimadzu LC–MS 8040 model mass spectrometer with an ESI source that could operate in triple and quadrupole modes in addition to negative and positive ionization modes was used to examine the phytochemical conditions. The data collection and processing were managed using Lab Solutions software (Shimadzu, Kyoto, Japan) via the multiple reaction monitoring (MRM) mode. Three separate analyses of each chemical were carried out: the first for quantitative assessment and the second and third for validation.

A number of critical mass spectrometer parameters were adjusted, including the DL temperature (250°C), the interface temperature (350°C), the heatsink temperature (400°C), the dryer gas flow rate (15 L/min), and the nebulizer gas flow rate (3 L/min). The formula used to calculate the results was Standard Deviation (±) = Analyte Value Result × U Value/100 after the standard deviation values were incorporated into the LC–MS/MS analysis (Aydemir, Arslan, et al., 2024; Yilmaz, 2020).

### 2.2 Preparation of meat samples

The marinade ingredients used for marinating red meat, chicken meat, and fish meat samples were obtained from a local market in Şanlıurfa, Türkiye. For red meat, beef (musculus longissimus thoracis) was utilized, chicken breast meat (musculus pectoralis) for chicken meat, and rainbow trout (*Oncorhynchus mykiss*) fillets for fish meat. Standardizing to a thickness of 1 cm for sirloin, chicken, and fish fillets, they were portioned into 100 g each. The marinade, used for marinating the meat samples, consisted of 20% tomato paste, 20% pepper paste, 20% olive oil, 4.5% salt, 7% garlic, 1.5% red pepper, 1% black pepper, 1% cumin, 10% natural lemon juice, and 15% yogurt. Dried and floured *M. fruticosa* was added to the marinade at 1% and 2% (1 and 2 g per 100 g marinade). Notably, *M. fruticosa* was omitted in the marinade for the control groups. The selection of these ingredients and their proportions in the marinade, as well as the determination of the *M. fruticosa* ratios, were based on thorough sensory evaluations in preliminary testing. All the ingredients were blended for 3 min using a blender (Korkmaz A457 Mia Multi 1300 W Blender) to ensure a homogeneous mixture. The mixture obtained was poured over the prepared meat samples and applied to cover the entire surface. The ratio of meat to marinade solution was 1:2. After marination, each group of meat samples was placed in separate foam trays (Plastic white foam, model no: 20 × 15 × 2.5 cm, China) and packed in polyethylene film (Transparent, 45 × 300 m, 12 Micron, Roll‐Up, Bursa, Türkiye). These packaged meat samples were then stored at 4 ± 1°C for 24 h. A total of 72 meat samples were prepared for each meat type (3 groups × 2 meat samples for each group × 2 cooking methods × 2 cooking temperatures × 3 independent replicates). Meat samples for each meat type were randomly divided into three groups: the control group treated with marinade without *M. fruticosa*, one group with marinade containing 1% *M. fruticosa*, and the other group with marinade containing 2% *M. fruticosa*. Subsequently, random meat samples were taken for each group and each meat type and the prepared samples were cooked.

### 2.3 Cooking conditions for the meats

After the designated storage period (4 ± 1°C for 24 h), the meat samples were removed from their packaging and promptly subjected to the cooking process. Cooking was conducted using both an electric oven (Arçelik, MF 209, Turkey) and an air fryer (Philips HD9252/90). Cooking temperatures and durations of 200°C for 12 min and 250°C for 8 min were maintained. These selected cooking parameters were determined through preliminary trials to achieve the desired level of doneness and ensure the attainment of an internal temperature of 75°C in the meat samples. A preheating duration of 10 min was observed before commencing the cooking process for both the oven and air fryer. To prevent the influence of external oil on oxidation, neither solid nor liquid oil was utilized during the cooking procedure. To monitor the cooking temperature, both in the air fryer and oven, and the internal temperature of the samples, K‐type thermocouples (HI 9057 KJT Thermocouple, Hanna Instruments, Portugal) were employed throughout the cooking duration. The cooking time was accurately measured using a laboratory‐type stopwatch. Following the cooking process, the meat samples underwent pH determination, color analysis, TBARS analysis, CML, and CEL analysis. In addition, dry matter, pH, fat, and protein contents of unmarinated raw meat samples were determined. Each analysis was conducted in duplicate and replicated three times (Aydemir, Arslan, et al., 2024).

### 2.4 Analysis of meat samples

#### 2.4.1 Physicochemical analysis of meat samples

To evaluate pH, 5 g of the sample was blended with 45 mL of distilled water, and the pH was gauged using a pH meter adhering to AOAC guidelines (Association of Analytical Chemists [AOAC], 1990). Protein content was assessed through the Kjeldahl method, following AOAC protocols (AOAC, 1998), while fat content was determined using the Soxhlet extraction technique outlined in AOAC (AOAC, 2002a). The dry matter was determined by the gravimetric method (AOAC, 2002b).

#### 2.4.2 Determination of thiobarbituric acid reactive substances (TBARS) in meat samples

A commercial ELISA kit (Oxford Biomedical Research, Food TBARS Assay Kit/FS50, USA) was used to analyze the samples for TBARS. Using an IKA T 25 Digital Ultra‐Turrax® Homogenizer, meat samples were homogenized. After homogenate was removed, about 25 ± 1 mg was put into 1.5 mL centrifuge tubes. To obtain the supernatants, 250 μL of RIPA buffer (INtRON Biotechnology) was added to each tube. The tubes were then centrifuged at 1600 × *g* for 10 min at 4°C.

The vial tubes were labeled and then filled with 100 μL of each sample. Each tube was then filled with 100 μL of sodium dodecyl sulfate (SDS) solution and 4 mL of color reagent that contained TBA acetic acid solution, TBA NaOH, and TBA thiobarbituric acid (TBA). For an hour, the tubes were cooked in a water bath. The samples were allowed to cool to room temperature on ice for 10 min after they had boiled in order to stop the reaction.

After a 10‐min centrifugation at 1600 × *g* and 4°C, the samples were allowed to come to room temperature for half an hour. After this incubation time, 150 μL of each sample was added to the ELISA plate, and a microplate reader (Wallac Microplate Spectrometer 1420‐011) was used to determine the absorbance readings at a wavelength of 530–540 nm. The samples' TBARS levels were expressed as mg MDA/kg (Aydemir et al., 2023; Aydemir, Arslan, et al., 2024).

#### 2.4.3 Determination of N^ε^‐(carboxyethyl)lysine (CEL) and N^ε^‐(carboxymethyl)lysine (CML) in meat samples

The analysis of CEL and CML in meat samples was conducted using the OxiSelect^TM^ N^ε^‐(carboxyethyl)lysine (CEL) and OxiSelect^TM^ N^ε^‐(carboxymethyl)lysine (CML) Competitive ELISA kit (Aydemir, Arslan, et al., 2024). To prepare the samples, cooked meat samples underwent homogenization using an IKA T 25 Digital Ultra‐Turrax® Homogenizer, followed by dilution with 1:10 w/v phosphate‐buffered saline (PBS). To ensure protein denaturation, the mixtures were stored at −80°C for 6 h. Subsequently, a 0.2% proteinase K solution (Sigma, 20 mg/mL) was added to the samples, which were then left to incubate at 37°C overnight. The enzyme deactivation process involved heating the mixtures for 10 min at 80°C. The supernatants were obtained by centrifugation at 1600 × *g* for 10 min at 4°C.

The ELISA study was conducted using a 96‐well ELISA plate. Each well received 100 μL of 1× CEL or CML conjugate, which was prepared using coating buffer (pH 9.6, 0.02% NaN_3_, and 0.1 M NaHCO_3_). After being incubated at 4°C for 24 h, the plate was dried and subsequently rinsed three times with 200 μL of PBS containing 0.05% Tween‐20, w/v, and 1 mM NaN_3_. The assay diluent (200 μL) was then added to each well and the mixture was shaken at room temperature for 1 h (using a Biosan MR‐12 shaker).

The wells coated with CEL and CML conjugate were then filled with the prepared samples and 50 μL of CEL‐BSA or CML‐BSA standard. Following that, they were incubated on an orbital shaker for 5 min at room temperature. Next, 50 μL of diluted anti‐CEL or anti‐CML antibody was added to each well, and the mixture was incubated at room temperature for an hour using an orbital shaker. After being cleaned three times using 250 μL of 1× wash buffer, the wells were dried. Subsequently, each well was filled with 100 μL of diluted secondary HRP‐conjugated antibody, which was then incubated for an hour at room temperature on an orbital shaker. Following a third round of washing the wells with 250 μL of 1× wash buffer and drying, each well received 100 μL of substrate solution. The wells were then incubated at room temperature on an orbital shaker for 2–20 min, or until a color shift was observed.

After adding 100 μL of stop solution to each well, a microplate reader (Wallac Microplate Spectrometer 1420‐011) was used to measure the absorbance values at 450 nm. The computations were carried out by creating graphs using the data from the control wells, and the findings were expressed in μg/g (Aydemir, Arslan, et al., 2024).

#### 2.4.4 Color analysis of meat

Using a CS‐10° 8 mm portable digital colorimeter (Tuodapu, Inc., China), the color of fresh beef was measured. The colorimeter was calibrated using standard white tiles before each measurement session. Hunter settings, which were set to a 10° standard observer angle and D‐65 illuminant type, were used to assess the samples' attributes of L* (whiteness/darkness), a* (redness/greenness), and b* (yellowness/blueness). Each beef sample was measured at three different locations on its surface, and a 30‐min blooming period was applied (Aydemir, Arslan, et al., 2024; Hunt et al., 1991).

### 2.5 Statistical analysis

Three separate and independent replicates were used for the analysis of the plant characteristics and evaluations of the meat samples. The dataset resulting from the study of meat sample compositions, color, pH, TBARS, CEL, and CML, as well as TPC, TFC, ABTS, DPPH tests, and phenolic compounds, is shown as mean ± standard error. All gathered data were tested for homogeneity using the Levene test and normalcy using the Kolmogorov–Smirnov test prior to analysis. Plant characterization (including TPC, TFC, ABTS, DPPH tests, and bioactive phytochemicals) was done using descriptive statistics.

The general linear model (GLM) was utilized for the statistical examination of color, pH, TBARS, CEL, and CML data. The GLM procedure was conducted with the equation as specified. In the GLM procedure, the cooking method (air fryer and oven), cooking temperatures (200 and 250°C), and *M. fruticosa* level (0%, 1%, and 2%) were identified as fixed effects, while replicates were designated as random effects. Multiple comparisons were performed utilizing Duncan's test (*p* < .05). The experiment was replicated three times, and each repetition was executed at different instances employing diverse raw materials.

## 3 RESULTS

### 3.1 Total phenolic contents (TPC) and total flavonoid contents (TFC) of *M. fruticosa*

TPC and TFC of *M. fruticosa* extracts were determined, and the results are presented in Table 1.

**TABLE 1:** TPC and TFC values of *Micromeria fruticosa* extracts (Mean ± SE).

Extract type	TPC (mg gallic acid equivalent/g)	TFC (mg quercetin equivalent/g)
Water extract	5.17 ± 0.42^b^	16.17 ± 0.64^b^
Methanol extract	7.23 ± 0.07^a^	28.42 ± 1.15^a^

*Note*: a and b: Statistically significant differences are shown by values in the same column that have distinct superscripts (*p* < .05).

### 3.2 Antioxidant activity of *M. fruticosa*

The DPPH and ABTS scavenging activities of *M. fruticosa* are presented in Table 2.

**TABLE 2:** DPPH and ABTS activity levels of *Micromeria fruticosa* extracted by methanol and water (mean ± SE).

	Concentration amounts (μg/mL)	Water extract	Methanol extract
ABTS	50	57.87 ± 3.45^Be^	98.24 ± 5.76^Ae^
	100	79.51 ± 2.47^Bd^	129.92 ± 7.38^Ad^
	250	133.10 ± 3.59^Bc^	187.08 ± 2.65^Abc^
	500	252.29 ± 3.24^Bb^	290.82 ± 6.12^Aab^
	1000	287.75 ± 3.37^Ba^	300.74 ± 2.1^Aa^
DPPH	50	24.75 ± 1.79^Bcd^	53.56 ± 2.81^Ae^
	100	28.09 ± 2.37^Bc^	77.24 ± 3.56^Ad^
	250	62.63 ± 2.87^Bc^	115.74 ± 4.06^Ac^
	500	139.32 ± 6.31^Bb^	211.62 ± 6.64^Ab^
	1000	232.46 ± 6.91^Ba^	266.13 ± 8.26^Aa^

*Note*: A–C: In the same row, values with distinct superscripts indicate statistical differences (*p* < .05). a–f: In the same column, values with distinct superscripts indicate statistical differences (*p* < .05).

### 3.3 Bioactive phytochemicals of *M. fruticosa*

**FIGURE 1:**
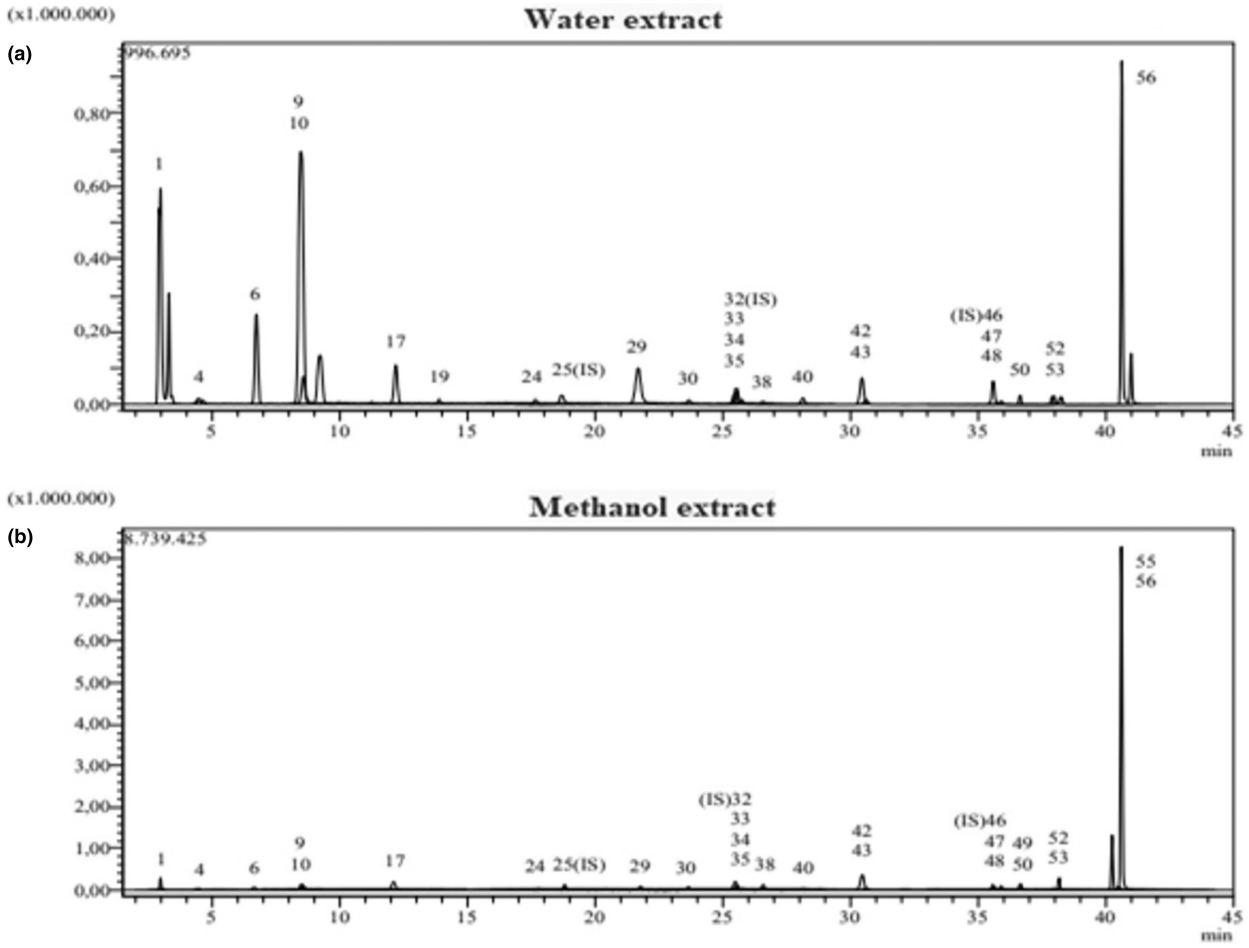
LC‐MS/MS chromatograms of water and methanol extract of *Micromeria fruticosa*. (a) The chromatogram of the water extract (b). The chromatogram of methanol extract.

The LC–MS/MS outcomes for *M. fruticosa* are detailed in Table 3. Additionally, Figure 1a illustrates the chromatogram of the water extract, while Figure 1b displays the chromatogram of the methanol extract. Validation outcomes for each compound can be found in Table S1, and the standard chromatogram is depicted in Figure S1.

**TABLE 3:** Phenolic compounds of *Micromeria fruticosa* (μg analyte/L ± uncertainty).

Number in chromatography	Analytes	Water extract	Methanol extract
1	Quinic acid	91,048 ± 3.386	17,424 ± 0.648
4	Gallic acid	396 ± 0.004	464 ± 0.005
6	Protocatechuic acid	6614 ± 0.231	2313 ± 0.080
9	Chlorogenic acid	17,944 ± 0.382	10,503 ± 0.223
10	Protocatechuic aldehyde	12,100 ± 0.004	443 ± 0.017
17	Caffeic acid	635 ± 0.009	1462 ± 0.022
19	Vanillin	59 ± 0.007	ND
24	p‐Coumaric acid	240 ± 0.004	269 ± 0.005
29	Salicylic acid	569 ± 0.008	616 ± 0.009
30	Cyranoside	221 ± 0.008	564 ± 0.020
33	Rutin	2010 ± 0.004	6110 ± 0.150
34	Isoquercitrin	2008 ± 0.044	9294 ± 0.204
35	Hesperidin	564 ± 0.001	1989 ± 0.066
38	Rosmarinic acid	194 ± 0.002	6186 ± 0.080
40	Cosmosiin	480 ± 0.000	1423 ± 0.011
42	Astragalin	3203 ± 0.036	16,901 ± 0.192
43	Nicotiflorin	636 ± 0.006	2219 ± 0.023
47	Quercetin	82 ± 0.000	193 ± 0.003
48	Naringenin	4 ± 0.000	534 ± 0.020
49	Hesperetin	ND	7 ± 0.000
50	Luteolin	24 ± 0.000	2320.007
52	Kaempferol	46 ± 0.000	62 ± 0.001
53	Apigenin	43 ± 0.000	529 ± 0.009
55	Chrysin	N.D.	30 ± 0.00
56	Acacetin	5240 ± 0.019	46,722 ± 1.696

Abbreviation: ND, Not detected.

### 3.4 Composition of meat samples

The composition and pH of the meat samples used in the analyses are provided in Table 4.

**TABLE 4:** Dry matter, fat, protein, and pH of raw meat samples (mean ± SE).

	Dry matter (%)	Fat (%)	Protein (%)	pH
Beef	34.87 ± 1.13	13.6 ± 0.42	22.3 ± 0.65	5.63 ± 0.15
Chicken meat	28.6 ± 1.03	3.4 ± 0.32	24.5 ± 0.72	5.78 ± 0.18
Fish meat	24.4 ± 1.48	2.8 ± 0.06	20.17 ± 0.49	6.10 ± 0.27

### 3.5 Color values of meat samples

Table 5 displays the L*, a*, and b* values for the meat groups. The L* value of fish meat varied statistically significantly (*p* < .05) between the groups. There was a statistically significant difference (*p* < .05) in the a* values across the groups of beef meat. Every type of meat disclosed a statistically significant difference (*p* < .05) between the groups, which was consistent with the b* value.

**TABLE 5:** Color levels of meat samples (Mean ± SE).

Color parameter	Groups	Beef	Chicken meat	Fish meat
L*	Control	33.75 ± 4.44	41.44 ± 1.95	41.2 ± 1.41^b^
	*Micromeria fruticosa* 1%	37.68 ± 4.46	40.34 ± 1.20	45.76 ± 2.46^a^
	*M. fruticosa* 2%	30.27 ± 3.92	37.41 ± 3.63	41.74 ± 1.88^b^
	Unmarinated meat group	32.70 ± 2.57	39.94 ± 5.65	45.23 ± 2.00^a^
a*	Control	27.92 ± 5.99^ab^	11.45 ± 3.83	19.13 ± 5.54
	*M. fruticosa* 1%	20.24 ± 4.70^b^	11.61 ± 2.67	18.01 ± 3.62
	*M. fruticosa* 2%	22.02 ± 5.82^b^	11.27 ± 2.95	20.32 ± 3.82
	Unmarinated meat group	37.65 ± 3.51^a^	6.99 ± 3.00	16.30 ± 1.58
b*	Control	15.80 ± 2.28^b^	20.63 ± 1.36^b^	27.24 ± 5.15^b^
	*M. fruticosa* 1%	17.84 ± 3.15^ab^	23.28 ± 3.80^b^	35.67 ± 3.11^a^
	*M. fruticosa* 2%	22.90 ± 5.41^a^	30.35 ± 4.99^a^	35.48 ± 1.67^a^
	Unmarinated meat group	5.72 ± 1.45^c^	9.57 ± 1.26^c^	14.25 ± 1.17^c^

*Note*: *M. fruticosa*: *M. fruticosa*, 1% or 2%: Amount of *M. fruticosa* added to marinade, L* (brightness), b* (yellow‐blue), a* (red‐green), a–c: In the same column, values with distinct superscripts indicate statistical differences (*p* < .05).

### 3.6 pH value of meat samples

**FIGURE 2:**
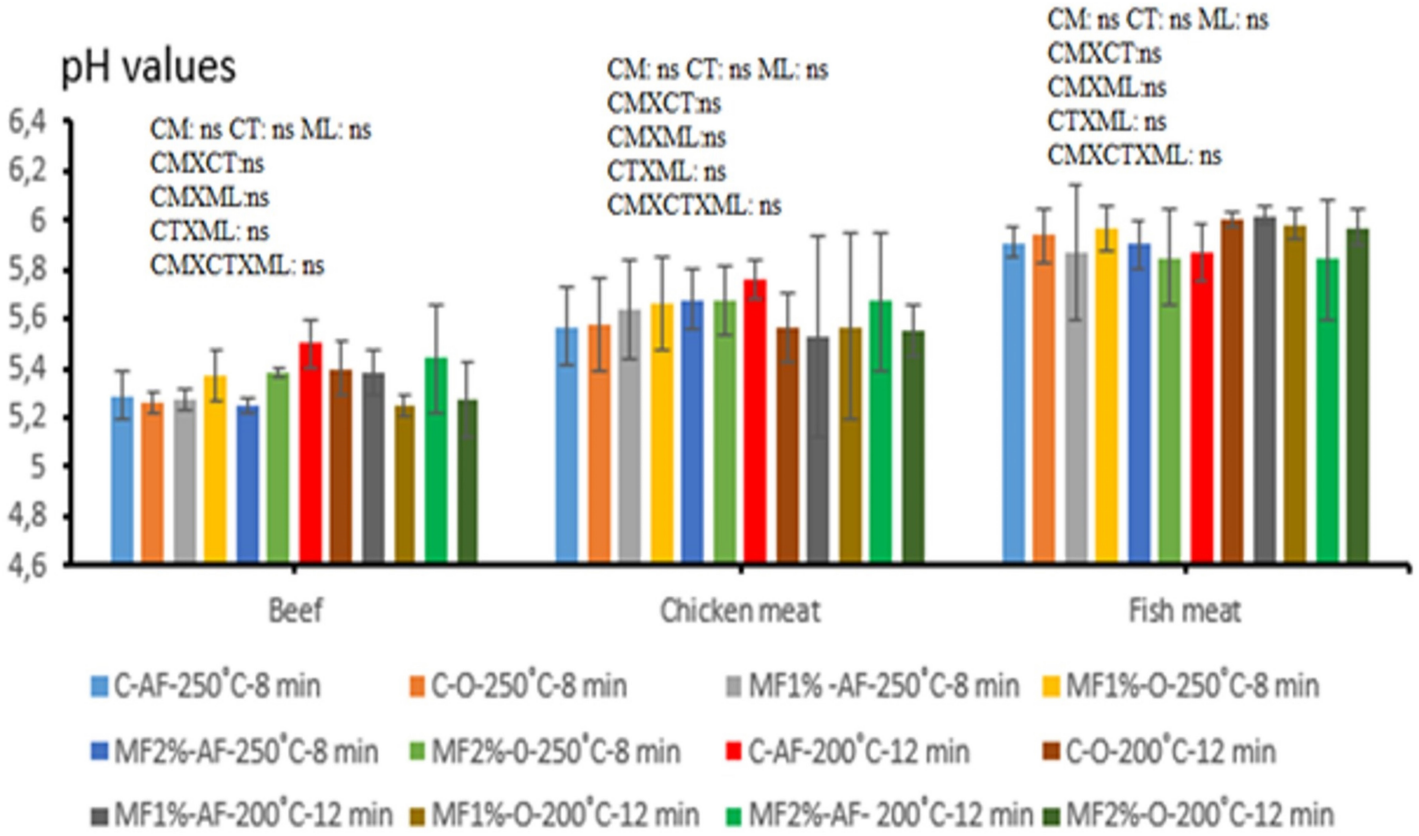
pH levels of meat samples (Mean ± SE). 1% or 2%: Amount of *Micromeria fruticosa* added to marinade; AF, Air frying; C, Control; CM, Cooking method; CT, Cooking temperature; MF, *M. fruticosa*; Min, Minute; ML, *M. fruticosa* level; ns, not significant; O, Oven frying.

The pH values of the meat samples are given in Figure 2.

### 3.7 TBARS value of meat samples

**FIGURE 3:**
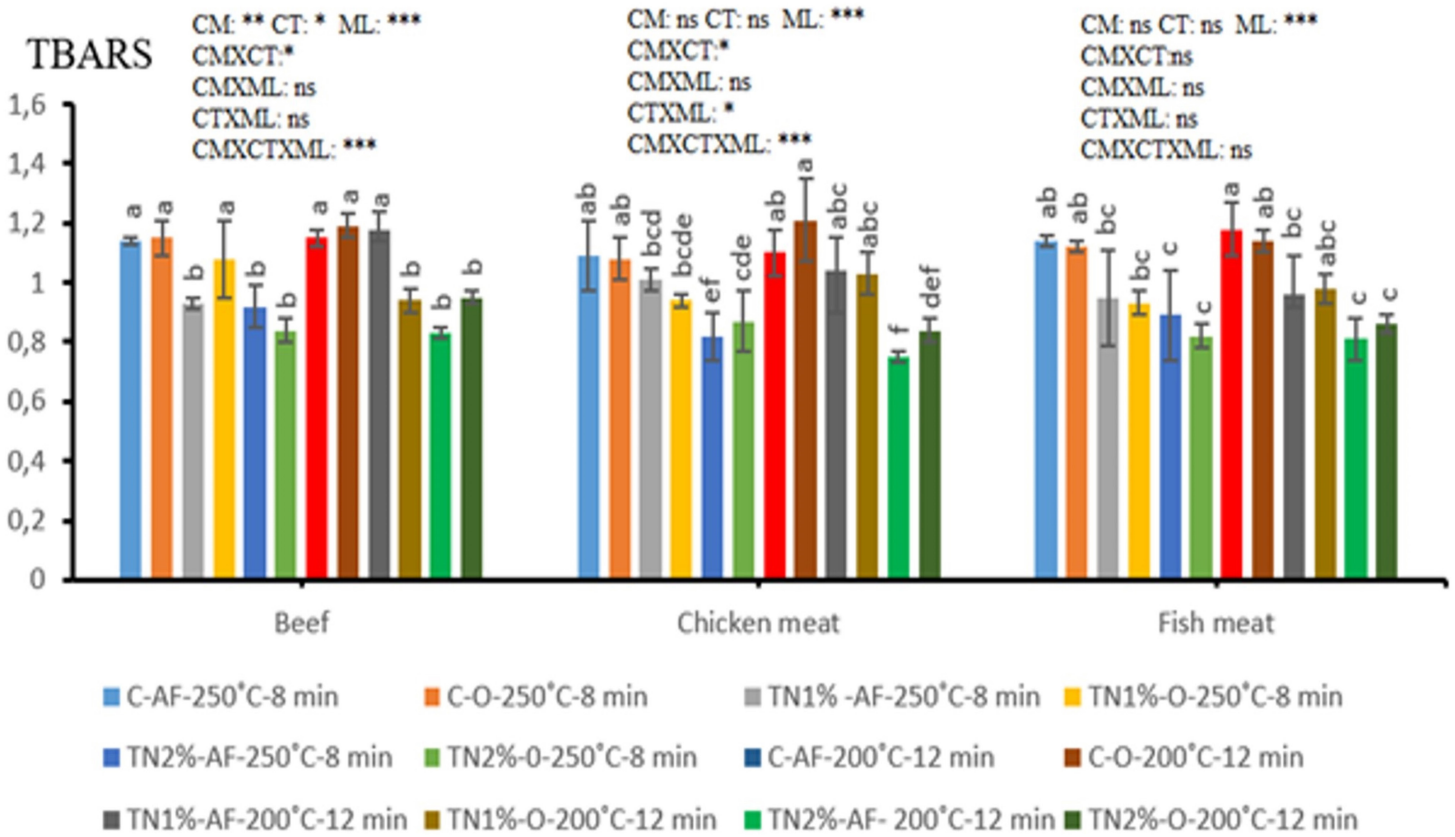
TBARS levels of meat samples (mean ± SE). 1% or 2%: Amount of *Micromeria fruticosa* added to marinade; AF, Air frying; C, Control; CM, Cooking method; CT, Cooking temperature; MF, *M. fruticosa*; Min, Minute; ML, *M. fruticosa* level; ns, not significant; O, Oven frying. a–f: The mean values with different letters between the groups are statistically different (*p* < .05). *p < .05, **p < .01, ***p < .001.

The TBARS values of the meat samples are given in Figure 3.

### 3.8 N^ε^‐(carboxyethyl)lysine (CEL) and N^ε^‐(carboxymethyl)lysine (CML) values of meat samples

The CML values of meat groups are presented in Table 6. The CEL values of meat groups are presented in Table 7.

**TABLE 6:** Interaction effects and ANOVA analysis results for CML (mean (μg/g) ± SE).

Marination groups (*Micromeria fruticosa* level)	Cooking method	Cooking temperature (°C)	Beef	Chicken meat	Fish meat
Control (0%)	AF	200	16.99 ± 0.14^a^	16.57 ± 0.28^a^	5.12 ± 0.86^a^
	AF	250	15.62 ± 0.28^bc^	16.29 ± 0.72^ab^	4.57 ± 0.32^a^
	O	200	13.98 ± 0.70^de^	15.46 ± 0.30^ab^	5.12 ± 0.32^a^
	O	250	13.88 ± 0.48^def^	12.12 ± 0.14^ef^	3.02 ± 0.25^b^
1%	AF	200	15.84 ± 0.19^b^	11.21 ± 0.10^fg^	2.44 ± 0.48^bcd^
	AF	250	12.86 ± 0.58^g^	10.50 ± 0.11^g^	2.76 ± 0.11^bc^
	O	200	15.04 ± 0.31^bc^	14.02 ± 0.86^cd^	3.30 ± 0.14^b^
	O	250	15.75 ± 0.19^b^	15.04 ± 0.79^bc^	1.58 ± 0.34^de^
2%	AF	200	14.75 ± 0.53^cd^	13.51 ± 0.14^d^	1.72 ± 0.34^cde^
	AF	250	13.91 ± 0.18^def^	13.05 ± 0.66^de^	1.30 ± 0.05^e^
	O	200	13.52 ± 0.43^efg^	10.05 ± 0.74^g^	2.73 ± 0.14^e^
	O	250	13.91 ± 0.18^def^	13.05 ± 0.66^de^	1.30 ± 0.05^e^
Statistics					
CM			***	***	***
CT			***	***	***
ML			***	***	***
CMXCT			***	*	***
CMXML			***	***	***
CTXML			**	***	***
CMXCTXML			***	***	**

*Note*: a–g: In the same column, values with distinct superscripts indicate statistical differences (*p* < .05).

Abbreviations: AF, Air frying; CM, Cooking method; CMXCT, Interaction between cooking method and cooking temperature; CMXCTXML, Interaction among cooking method, cooking temperature, and *M. fruticosa* level; CMXML, Interaction between cooking method and *M. fruticosa* level; CT, Cooking temperature; CTXML, Interaction between cooking temperature and *M. fruticosa* level; Min, Minute; ML, *M. fruticosa* level; O, Oven frying.

*p < .05, **p < .01, ***p < .001.

**TABLE 7:** Interaction effects and ANOVA analysis results for CEL (mean (μg/g) ± SE).

Marination groups (*Micromeria fruticosa* level)	Cooking method	Cooking temperature (°C)	Beef	Chicken meat	Fish meat
Control (0%)	AF	200	17.66 ± 0.05^a^	15.08 ± 0.87^a^	17.61 ± 0.16
	AF	250	17.59 ± 0.26^a^	14.34 ± 0.65^a^	17.43 ± 0.19
	O	200	17.73 ± 0.09^a^	12.77 ± 0.71^b^	17.39 ± 0.26
	O	250	17.60 ± 0.28^a^	12.06 ± 0.72^bc^	17.32 ± 0.31
1%	AF	200	17.38 ± 0.26^a^	10.98 ± 0.16^cd^	17.33 ± 0.30
	AF	250	17.44 ± 0.30^a^	9.18 ± 0.13^d^	17.38 ± 0.23
	O	200	17.39 ± 0.24^a^	9.51 ± 0.16^d^	17.38 ± 0.27
	O	250	8.97 ± 0.32^d^	5.96 ± 0.61^g^	17.24 ± 0.32
2%	AF	200	15.43 ± 0.32^b^	10.23 ± 0.64^de^	17.38 ± 0.50
	AF	250	13.87 ± 0.83^c^	9.04 ± 0.22^df^	17.26 ± 0.33
	O	200	9.43 ± 0.80^d^	7.75 ± 0.63^f^	17.29 ± 0.35
	O	250	8.97 ± 0.32^d^	5.96 ± 0.61^g^	17.24 ± 0.32
Statistics					
CM			***	***	ns
CT			***	***	ns
ML			***	***	ns
CMXCT			ns	*	ns
CMXML			***	***	ns
CTXML			***	**	ns
CMXCTXML			**	***	ns

*Note*: a–g: In the same column, values with distinct superscripts indicate statistical differences (*p* < .05).

Abbreviations: AF, Air frying; CM, Cooking method; CMXCT, Interaction between cooking method and cooking temperature; CMXCTXML, Interaction between cooking method, cooking temperature, and *M. fruticosa* level; CMXML, Interaction between cooking method and *M. fruticosa* level; CT, Cooking temperature; CTXML, Interaction between cooking temperature and *M. fruticosa* level; Min, Minute; ML, *M. fruticosa* level; ns, not significant; O, Oven frying.

*p < .05, **p < .01, ***p < .001.

## 4 DISCUSSION

### 4.1 Total phenolic contents (TPC) and total flavonoid contents (TFC) of *M. fruticosa*

The comparison between the methanol and water extracts revealed higher levels of TPC and TFC in the methanol extract, as depicted in Table 1. This is thought to be due to methanol being a better solvent for organic acids than water (Pól et al., 2007). As a matter of fact, Takım (2020) reported that methanol is a better solvent than water. Moreover, it was observed that the TFC content exceeded the TPC content. These findings suggest a substantial presence of bioactive compounds in *M. fruticosa*. However, our results slightly deviate from those reported for TPC and TFC of *M. fruticosa* (Al‐Shboul et al., 2022). These discrepancies could be caused by a number of reasons, such as the growth conditions of the plant, environmental factors (such as climate and geography), the time of harvest, the conditions of postharvest, extraction techniques, solvents used in extraction, sample preparation techniques, and the variety of analytical methodologies used (Rodríguez‐Carrasco et al., 2018).

### 4.2 Antioxidant activity of *M. fruticosa*

The investigation revealed notable antioxidant activities in *M. fruticosa*, as demonstrated in Table 2. This behavior can be explained by *M. fruticosa*'s higher concentration of bioactive chemicals. Indeed, the established role of phenolics and flavonoids in antioxidant capacity is well documented (Al‐Nuri et al., 2022). Previous research has also highlighted the significant antioxidant activity of *M. fruticosa*, although the outcomes of this study may exhibit varying activity levels (Al‐Nuri et al., 2022; Al‐Shboul et al., 2022; Azab, 2023). These observed discrepancies are likely attributable to differences in the composition of bioactive compounds within *M. fruticosa*, resulting in variations in their bioactive properties. Furthermore, variations in ABTS and DPPH concentrations, standard substance concentrations, and spectroscopic methodologies could significantly contribute to these disparities (Seyhan, 2019).

### 4.3 Bioactive phytochemicals of *M. fruticosa*

The methanol extract of *M. fruticosa* revealed the presence of 24 phenolic compounds, while the water extract contained 23 phenolic compounds, as indicated in Table 3. Notably, the water extract exhibited higher levels of quinic acid (91,048 ± 3.386 μg/analyte/g), chlorogenic acid (17,944 ± 0.382 μg/analyte/g), and protocatechuic aldehyde (12,100 ± 0.004 μg/analyte/g). Conversely, the methanol extract showcased elevated levels of quinic acid (17,424 ± 0.648 μg/analyte/g), astragalin (16,901 ± 0.192 μg/analyte/g), chlorogenic acid (10,503 ± 0.223 μg/analyte/g), and isoquercitrin (9294 ± 0.204 μg/analyte/g). This investigation underscores the richness of phenolic compounds in *M. fruticosa*, with both water and methanol extracts exhibiting abundant quantities of quinic acid and chlorogenic acid compounds (Table 3). In line with these findings, a research conducted by Abu‐Reidah et al. (2019) reported a similar abundance of phenolic compounds, emphasizing the high levels of chlorogenic acid and quinic acid present in *M. fruticosa*. It has also been documented that the plant contains more than 180 phytochemicals, including 87 flavonoids, 41 phenolic acids, 16 terpenoids, 8 sulfate derivatives, 7 iridoids, and others. Another study found that the average total phenolic content of the plant's pollen was 56.78 ± 0.49 mg gallic acid equivalent (GAE) per gram, while flavone and flavonol content ranged from 2.48 ± 0.05 to 8.03 ± 0.01 mg quercetin equivalent (QE) per gram (Sadeq et al., 2021). El Hamaoui et al. (2015) reported that the major bioactive compounds in the ethanolic extract of *M. fruticosa* grown in Lebanon were chlorogenic acid, naringenin, quercetin, and ellagic acid. The slight variation in the mint content from the reported results may be due to differences in several reasons, including, as mentioned above, the growing conditions of the plant used in the analysis, environmental factors (such as climate and geography), harvest time, postharvest conditions and extraction techniques, and solvents used in the extraction (Rodríguez‐Carrasco et al., 2018).

### 4.4 Composition of meat samples

It is important to remember that the dry matter, protein, and fat composition of beef meat can vary within the ranges 20%–35%, 16%–22% and 1.5%–13%, respectively. Similarly, chicken meat may exhibit dry matter, protein, and fat content ranging between 26% and 46%, 17% and 23.29%, and 1.2% and 4.5%, respectively, while fish meat can range between 16% and 34% for dry matter, 15% and 24% for protein, and 0.1% and 22% for fat (Arslan, 2022). In a study by Chen and Scott Smith (2015), the protein content of raw beef, chicken, and fish meat was reported as 21.53%, 21.88%, and 18.59%, respectively, with fat content recorded as 7.18%, 4.25%, and 1.94%, respectively, and with moisture content recorded as 69.25%, 73.92%, and 75.03%, respectively. The pH values were reported as 5.56, 6.16, and 6.85 for beef, chicken, and fish meat, respectively, in their investigation of advanced glycation end products in cooked meat products. Sun et al. (2016) reported protein levels ranging from 16.50% to 22.25% for beef and 18.6%–24.2% for chicken breast samples. Additionally, Aydemir et al. (2023) documented pH values for beef minced meat ranging between 5.66 and 6.06 on day 0 of storage in their study on CML inhibition in meats. In another study exploring the effects of aging, storage, and heat treatment on the formation of CML in chicken meat, the pH of chicken breast was reported as 6.09, with a decrease to 5.81 observed after an increase in aging and storage time to 6 h (Huang et al., 2022). These reported results closely align with the findings of the present study. Any minor discrepancies observed are likely attributed to factors such as meat freshness, hygiene conditions, rigor mortis development, and the breed, age, and diet of the animal, all of which can influence the composition and physicochemical properties of the meat.

### 4.5 Color values of meat samples

The L* value in meat and meat products can exhibit variability due to factors such as composition, pigment type and concentration, fiber content, moisture levels, and air absorption (Viuda‐Martos et al., 2010). In this investigation, it was observed that *M. fruticosa* exerted a statistically significant influence solely on the L* value of fish meat, most likely due to the phenolic chemicals that *M. fruticosa* naturally contains. The measurement of the a* value holds particular significance in assessing oxidation levels in red meat and its derivatives (Kim et al., 2013). Interestingly, *M. fruticosa* demonstrated a significant impact solely on the a* value of beef meat, indicating a potential role of *M. fruticosa* pigments in attenuating the a* value. Additionally, *M. fruticosa* exhibited a statistically significant effect on the b* value across all meat types. Variations in meat product composition, oxidation states, and the incorporation of phenolic compound‐rich plants have been reported to influence alterations in the b* value (Turgut et al., 2017). However, there is a dearth of literature investigating the specific impact of *M. fruticosa* on the color parameters of food products.

### 4.6 pH value of meat samples

The pH value serves as a critical determinant of meat and meat product quality. As depicted in Figure 2, the pH values of the cooked meat samples were examined. Typically, postslaughter, the pH of meat tends to decline to a range of 5.6–6.2 within a few hours. In our investigation, pH values ranged from 5.25 to 5.50 in beef groups, 5.46 to 5.92 in chicken groups, and 5.67 to 6.02 in fish groups (Figure 2). Notably, the marination process appeared to induce a reduction in the pH value of beef and fish meat samples. This occurrence could be outlined by the existence of particular organic acids that come from the herbs and spices that are included in the marinade's recipe. Concerning the pH levels across all meat types, the mean values of all interaction effects (cooking method, cooking temperature, and *M. fruticosa* level) were uniform (*p* > .05). This consistency could be elucidated by the high buffering capacity inherent in proteins (İncili et al., 2020).

### 4.7 TBARS value of meat samples

Lipid oxidation represents a significant factor contributing to the quality deterioration of meat and meat products. The measurement of thiobarbituric acid reactive substances (TBARS) stands as a pivotal indicator of lipid oxidation, particularly following heat treatment. As illustrated in Figure 3, elevated TBARS values were observed across all meat groups, reflecting the oxidative processes affecting fatty acids induced by the applied heat treatment. In particular, the range of TBARS measurements for beef, chicken, and fish meat was 0.83–1.19 mg MDA/kg, 0.75–1.21 mg MDA/kg, and 0.81–1.18 mg MDA/kg, respectively (Figure 3). It is noted that the perceived minimum level of rancidity in meat and meat products by consumers is 0.5 mg MDA/kg (Greene & Cumuze, 1982) and the acceptable upper limit for MDA level is reported to be 1 mg/kg (Khorshidi et al., 2021). The fact that TBARS values exceeded the threshold of 0.5 mg MDA/kg in the current study can be explained by the fact that the heat treatment applied to meat samples accelerates the oxidation of fatty acids and the TBARS value varies depending on the measurement method. Indeed, Xu, Liu, et al. (2024) reported that malondialdehyde (MDA) is unstable at high temperatures and oxidizes further to form volatile compounds or reacts more rapidly with amino‐containing compounds after formation. In addition, the marinade composition used in the meats may also have influenced this. In fact, Jia et al. (2023) stressed that the compositions used in the curing of meats have an effect on oxidation, in particular, the use of salt in the composition is closely related to oxidation. Consistent with our findings, previous studies reported TBARS values for cooked meat ranging between 1.55 ± 0.21 and 1.78 ± 0.17 mg MDA/kg on day 0 and between 2.54 ± 0.28 and 3.28 ± 0.47 mg MDA/kg on day 16 (Aydemir et al., 2023). Another study employing the same method reported TBARS values for cooked meat on day 0 of storage ranging from 1.17 to 1.98 mg MDA/kg, and on day 16, they ranged from 1.70 to 3.34 mg MDA/kg (Aydemir, Arslan, et al., 2024).

The effect of *M. fruticosa* level was significant in all meats (*p* < .05), with TBARS values decreasing with the increase in *M. fruticosa* ratio in the marinade. The antioxidant qualities of the phenolic compounds found in the used plants are responsible for the observed effect (Zwolan et al., 2020). This result is consistent with similar investigations into how different plants and extracts affect the production of meat, which show reduced TBARS levels in groups using these supplements (Aydemir et al., 2023).

The interaction effects of Cooking method*Cooking temperature*M. fruticosa level were statistically significant for beef and chicken meat (*p* < .001), but not for fish meat (*p* > .05). Similarly, the mean interaction effects of Cooking method*Cooking temperature were statistically significant for beef and chicken meat (*p* < .001), but insignificant for fish meat (*p* > .05). This could be explained by the oxidation of fatty acids brought on by the meat's heat treatment (Aksu, 2009). These findings are consistent with previous studies reporting changes in TBARS values depending on the cooking process (Öztürk et al., 2023).

### 4.8 N^ε^‐(carboxyethyl)lysine (CEL) and N^ε^‐(carboxymethyl)lysine (CML) values of meat samples

The CEL and CML levels in meat groups are detailed in Tables 6 and 7, respectively. The CML amounts ranged from 12.86 to 16.99 μg/g in beef groups, 10.05 to 16.57 μg/g in chicken meat groups, and 0.63 to 5.12 μg/g in fish meat groups (Table 6). In comparison, the CEL amounts were found to be between 8.97 and 17.73 μg/g in beef groups, 5.96 and 15.08 μg/g in chicken meat groups, and 17.24 and 17.43 μg/g in fish meat groups (Table 7).

Studies by Aydemir, Arslan, et al. (2024) and Assar et al. (2009) reported CML levels in meat products cooked at varying temperatures and times, showing a wide range of values. Additionally, the CEL content in commercial meat products varied depending on the cooking method, with fried samples exhibiting higher levels compared to oven‐cooked ones (Yu et al., 2022). Also, studies exploring how different cooking techniques affect fish and chicken breast meat revealed varied quantities of CEL and CML, indicating that cooking situations have a major impact on the synthesis of these chemicals (Liu et al., 2022; Zhu et al., 2021).

The interaction effects of Cooking method*Cooking temperature were statistically significant for CML in beef, chicken meat, and fish meat, as well as for CEL in fish meat. This underscores the impact of cooking conditions on the formation of CEL and CML in meat samples. Previous studies have highlighted the contributions of cooking methods, temperature, and duration to the formation of these compounds (Chao et al., 2009; Goldberg et al., 2004; Huang et al., 2023). Many di‐carbonyl compounds, such as GO, which predispose to AGE formation during storage of raw meat, are produced by lipid oxidation. However, all of these reactions have little effect on the formation of AGEs during storage. However, it is emphasized that heat treatment greatly accelerates the formation of AGEs (Huang et al., 2023). It was therefore not surprising that the effect of cooking method and temperature on CML and CEL formation was significant.

The observed interaction effect of cooking method and cooking temperature in beef and fish meat is likely associated with the formation mechanism of CEL in meats, as suggested by Liu et al. (2017), who reported higher levels of methylglyoxal (MGO) (a precursor of CEL) than glyoxal (GO) (a precursor of CML) at high temperatures. This intricate interplay between cooking parameters and chemical reactions underscores the need for comprehensive understanding and control of cooking conditions to mitigate the formation of harmful compounds in meat products.

The means of the interaction effects for Cooking method*M. fruticosa level and Cooking temperature*M. fruticosa level were found to be statistically significant for CEL and CML in all meat types (Table 6). Specifically, for CEL, the means of the interaction effects for Cooking method*M. fruticosa level and Cooking temperature*M. fruticosa level were statistically significant for beef and poultry, but not for fish (Table 6). This observation may be explained by the bioactive substances present in *M. fruticosa*, which seem to disable the formation pathways of both CEL and CML. To reduce CEL and CML levels in food, it is suggested that, in addition to improving cooking methods, the addition of plants or plant extracts rich in polyphenols can be effective. In particular, it is believed that compounds such as quinic acid, chlorogenic acid, and protocatechuic acid, which are found in high amounts in concentrated cranberry juice, inhibit the formation of CML by binding to GO. Our findings are supported by Khalifa et al. (2020) reporting the inhibitory effect of quinic acid, and Tagliazucchi et al. (2019) reporting the inhibitory effect of protocatechuic acid. Additionally, compounds such as naringenin (Aydemir et al., 2023) and quercetin (Liu et al., 2017), rutin, hesperidin, catechin, quinic acid, isosercitrin, epigallocatechin, fumaric acid, 4‐OH benzoic acid, and kaempferol (Aydemir, Arslan, et al., 2024) are reported to inhibit AGE formation in foods.

In our study, an increase in the ratio of *M. fruticosa* in the marinade was found to inhibit CML formation, with the highest inhibition observed in fish meat. Similarly, the inhibition of CEL formation in beef and chicken meat was also observed with the increase in the ratio of *M. fruticosa* in the marinade. This inhibitory effect of CEL and CML formation by *M. fruticosa* is believed to be attributed to the presence of compounds such as quinic acid, chlorogenic acid, protocatechuic aldehyde, astragalin, and isoquercitrin, which are present in high amounts in the plant. Polyphenols are known to prevent the oxidation of free radicals produced by oxidation and also to bind dicarbonyl compounds, thereby blocking the formation pathways of CEL and CML (Lund & Ray, 2017). Additionally, the high antioxidant property of *M. fruticosa* is also thought to contribute to the inhibition of CEL and CML. Lipid oxidation during the preservation of meat and meat products has been found to impact the establishment of CEL and CML (Aydemir et al., 2023; Yu et al., 2018). In our study, a relationship between CEL and CML formation and TBARS was also observed. Consistent with our findings, other researchers have also reported a positive relationship between CML and oxidation (Aydemir, Arslan, et al., 2024; Öztürk et al., 2023). Additionally, Yu et al. (2018) reporting that there is a strong relationship between TBARS and CEL content also supports our findings. Data on the inhibition of CEL and CML by substances with polyphenol and antioxidant properties in meat and meat products are limited. Nevertheless, studies have emphasized the effectiveness of substances with polyphenol and antioxidant properties in inhibiting CEL and CML formation (Altun et al., 2024; Aydemir, Altun, et al., 2024; Aydemir, Arslan, et al., 2024; Chao et al., 2009; Zhu et al., 2021).

For CML, the means of the interaction effects of Cooking method*Cooking temperature*M. fruticosa level were statistically significant for beef (*p* < .001), chicken meat (*p* < .001), and fish meat (*p* < .01) (Table 6). The highest interaction effects were found in Control*Air frying*200°C for beef and chicken meat and in Control*Air frying*(200°C, 250°C) and Control*Oven frying*(200°C) for fish meat (Table 6). The lowest interaction effects in beef and chicken meat were found in the independent variables of 1% M. fruticosa*Air frying*250°C, 2% M. fruticosa*Oven frying*200°C, 2% M. fruticosa*Oven frying*(200°C, 250°C), and 2% M. fruticosa*Air frying*(200°C) in fish meat (Table 6). For CEL, the means of the interaction effects of Cooking method*Cooking temperature*M. fruticosa level were statistically significant for beef (*p* < .001) and poultry (*p* < .001), but not for fish (*p* > .05) (Table 7). The lowest interaction effects in beef and chicken meat were found in the independent variables 1% M. fruticosa*Oven frying*250°C and 2% M. fruticosa*Oven frying*(200°C, 250°C) (Table 7). Considering these findings, it is not surprising that the highest CML means were observed in the control group variables and the lowest means were observed in the variables containing *M. fruticosa*, especially in the variables containing 2% *M. fruticosa*. As mentioned above, the bioactive compounds contained in *M. fruticosa* can be explained by its inhibition of CEL and CML formation pathways. The detection of the lowest means for CEL and CML in the 250°C air frying independent variables clarified an important uncertainty.

The means of all interaction effects for CEL are the same for fish meat. According to this study, the amount of CEL was found to be high in all fish meat groups. This can be explained by the higher likelihood of myofibrillar proteins and glucose to produce CEL under thermal processing conditions (Zhang et al., 2022). Consistent with our study findings, it has been reported that the level of CEL in fried fish fillets is much higher than CML (Liu et al., 2022; Zhang et al., 2022). The CEL content in fish meat was found to be much higher than CML. This can be explained by the fact that methylglyoxal (MGO) (a precursor of CEL) is formed at higher levels than GO (a precursor of CML), and bioactive compounds present in *M. fruticosa* do not bind to GO formed at high levels, resulting in MGO. In fact, Liu et al. (2017) reported that the MGO content in the lysine–glucose system was approximately two times higher than GO under high‐temperature conditions. Furthermore, Srey et al. (2010) reported that MGO may have a higher reaction activity with lysine than GO during processing to produce CEL. Jiao et al. (2019) suggested that catechins significantly reduced the content of MGO, which is considered an important precursor of AGEs, but the catechins tested did not always inhibit CEL formation. This suggests that the main pathway of CEL formation may not always be related to MGO formation. Some bioactive compounds in *M. fruticosa* may slow down CEL inhibition and may not inhibit it. Taking all these explanations together, it seems reasonable that the CEL content in fish meat samples was higher compared to CML and that the interaction effects of cooking temperature methods and *M. fruticosa* were not significant. It also suggests that the pathways and factors influencing CML and CEL formation may be different.

The results of this study suggest that high temperature and prolonged cooking in the air fryer method can increase the formation of CEL and CML. Zhu et al. (2021) also reported that air fryer cooking of chicken meat reduced CEL and CML levels compared to deep frying. Similar to our findings in this study, it is emphasized that the application of low temperature and short cooking processes (3 min at 180°C) makes the air fryer cooking method advantageous.

It is known that there is a relationship between pH and CEL and CML levels (Vlassara & Uribarri, 2004). It is also emphasized that pH changes the physicochemical properties, solubility, and molecular conformation of macromolecules (Wang et al., 2022). It has been reported that CML formation can be inhibited more rapidly at low pH, whereas at high pH, the rate of MGO retention by phenolic and flavonoid compounds decreases, leading to slower inhibition of CML formation (Aydemir et al., 2023). The presence of more protons in the acidic state may help to reduce the total amount of free radicals and thus slow down lipid oxidation (Kim et al., 2016). Therefore, CML formation may be inhibited more rapidly due to the slowing of oxidation at low pH. Zhu et al. (2019) reported that the reaction rate between naringenin and methylglyoxal (MGO) is pH dependent. According to Yu et al. (2018), low pH was associated with the prevention of CEL and CML development. In the present study, this relationship was unclear because there was no statistically significant difference in pH between the meat groups. However, it can be emphasized that low levels of CEL and CML formation in meat samples may also be influenced by low pH values.

## 5 CONCLUSIONS

In conclusion, this study determined that *M. fruticosa* exhibits high total phenolic content, total flavonoid content, antioxidant activity, and a rich phenolic composition. The use of *M. fruticosa* in beef, chicken, and fish marinades had no negative effect on the color values of meat samples. It was determined that increasing the rate of *M. fruticosa* in the marinade was effective on the TBARS value, CEL, and CML formation. It was observed that CEL levels were significantly higher than CML levels in fish meat. The formation of CEL and CML in meat samples varied depending on the cooking method, applied temperature, and duration. For all control groups, those cooked in the air fryer had higher CML levels than those cooked in the oven, and higher CEL levels were observed with the air fryer cooking method, especially in chicken meat. The study revealed that the air fryer method, especially at high temperatures, might elevate the levels of CEL and CML, emphasizing the importance of choosing appropriate cooking methods and conditions for minimizing AGE formation. It should be important to consider that cooking methods and conditions (temperature, duration, pH, etc.) can influence the formation of CEL and CML. Therefore, selecting healthy cooking methods and using appropriate temperatures and durations are crucial in minimizing CEL and CML formation. Furthermore, the observed relationship between pH levels and CEL and CML formation adds a nuanced layer to the understanding of AGE dynamics in meat. While the impact of pH differences among meat groups in this study was not statistically significant, the low pH values could potentially contribute to the observed lower levels of CEL and CML formation.

The control of AGEs is a crucial aspect of nutrition and health. Finally, the potential of *M. fruticosa* to inhibit CEL and CML formation in meat samples is a significant finding for the food industry and consumer health. Further studies are warranted to explore the broader use and effects of this plant on a larger scale. By inhibiting the formation of detrimental AGEs, this natural ingredient offers a promising avenue for creating more healthful and palatable meat products. Further research exploring innovative ways to integrate *M. fruticosa* into various food preparations could pave the way for practical and impactful applications in both domestic and industrial settings.

Importantly, this research offers valuable insights for the food industry and consumers in the pursuit of healthier cooking practices to mitigate the formation of AGEs, contributing to improved food safety and nutritional outcomes. The study's detailed exploration of the interactions between cooking variables and the addition of *M. fruticosa* provides a solid foundation for future research aimed at refining cooking practices and optimizing the addition of natural compounds to enhance the nutritional quality of cooked meats.

## AUTHOR CONTRIBUTIONS

**Serap Kılıç Altun:** Conceptualization (equal); data curation (equal); formal analysis (equal); resources (equal); software (equal); writing – original draft (equal); writing – review and editing (equal). **Mehmet Emin Aydemir:** Conceptualization (equal); data curation (equal); formal analysis (equal); resources (equal); software (equal); writing – original draft (equal); writing – review and editing (equal). **Kasım Takım:** Data curation (equal); formal analysis (equal); software (equal); writing – original draft (equal); writing – review and editing (equal). **Mustafa Abdullah Yilmaz:** Formal analysis (equal); writing – original draft (equal).

## FUNDING INFORMATION

This study was supported by the Scientific Research Unit of Harran University under project number 21259.

## CONFLICT OF INTEREST STATEMENT

The authors declare no conflicts of interest.

## ETHICS STATEMENT

This article does not cover any human or animal studies conducted by any of the authors. Not applicable.

## Supporting information


Figure S1

Table S1

## Data Availability

The data are available upon request from the authors.
