# Supplementary figures and images for: Inhibition of Nε‐(carboxyethyl)lysine and Nε‐(carboxymethyl)lysine formation in beef, chicken, and fish meat: A comparative study of oven frying and air frying with a marinade‐containing Micromeria fruticosa
Source: Food Sci Nutr. 2024 Jun 14;12(9):6298–314. doi: 10.1002/fsn3.4276 (PMC11561824; doi:10.1002/fsn3.4276)

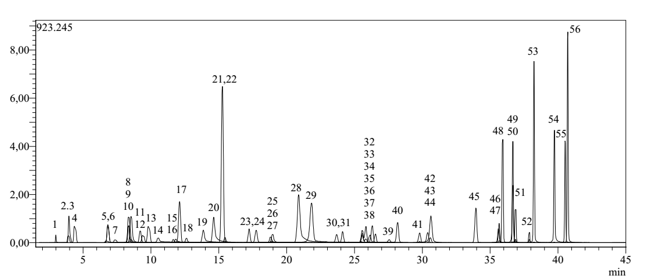


**Supplementary Figure 1.** LC-MS/MS chromatograms of water and methanol extract of *M. fruticosa*

Supplement: Supplementary file 1 — Figure S1 [file FSN3-12-6298-s001.docx]
